# Filariasis and lymphoedema

**DOI:** 10.1111/j.1365-3024.2009.01133.x

**Published:** 2009-11

**Authors:** K M PFARR, A Y DEBRAH, S SPECHT, A HOERAUF

**Affiliations:** 1 Institute for Medical Microbiology, Immunology and Parasitology, University Hospital BonnBonn, Germany; 2 Faculty of Allied Health Sciences, Kwame Nkrumah University of Science and TechnologyKumasi, Ghana; 3 Kumasi Centre for Collaborative Research in Tropical Medicine (KCCR)Kumasi, Ghana

**Keywords:** Filariasis, hydrocele, lymphedema, TLR, VEGF, *Wolbachia*

## Abstract

Among the causes of lymphoedema (LE), secondary LE due to filariasis is the most prevalent. It affects only a minority of the 120 million people infected with the causative organisms of lymphatic filariasis (LF), *Wuchereria bancrofti* and *Brugia malayi/timori*, but is clustered in families, indicating a genetic basis for development of this pathology. The majority of infected individuals develop filarial-specific immunosuppression that starts even before birth in cases where mothers are infected and is characterized by regulatory T-cell responses and high levels of IgG4, thus tolerating high parasite loads and microfilaraemia. In contrast, individuals with this pathology show stronger immune reactions biased towards Th1, Th2 and probably also Th17. Importantly, as for the aberrant lymph vessel development, innate immune responses that are triggered by the filarial antigen ultimately result in the activation of vascular endothelial growth factors (VEGF), thus promoting lymph vessel hyperplasia as a first step to lymphoedema development. *Wolbachia* endosymbionts are major inducers of these responses *in vitro*, and their depletion by doxycycline in LF patients reduces plasma VEGF and soluble VEGF-receptor-3 levels to those seen in endemic normals preceding pathology improvement. The search for the immunogenetic basis for LE could lead to the identification of risk factors and thus, to prevention; and has so far led to the identification of single-nucleotide polymorphisms (SNP) with potential functional relevance to VEGF, cytokine and toll-like receptor (TLR) genes. Hydrocele, a pathology with some similarity to LE in which both lymph vessel dilation and lymph extravasation are shared sequelae, has been found to be strongly associated with a VEGF-A SNP known for upregulation of this (lymph-)angiogenesis factor.

## Introduction

As a result of its heritable nature, much more is known about the molecular basis of primary lymphoedema (LE) in comparison with what is known about the aetiology and pathophysiology of secondary LE – caused by trauma, environmental factors (e.g. podoconiosis) or infection. The single largest source of secondary LE is lymphatic filariasis (LF), a disease caused by filarial nematodes (1).

An estimated 120 million people are infected with the filarial nematodes *Wuchereria bancrofti*, *Brugia malayi* and *Brugia timori*, which are the causative agents of LF, and 1·2 billion people are at risk of infection (2). The disease is found in sub-Saharan Africa, India, Southeast Asia, parts of South America, the Caribbean and the South Pacific. Adult filarial parasites are sexually dimorphic and reside in the lymphatic vessels, where they mate and produce thousands of first-stage larvae [microfilaria (MF)] for up to 8 years. Mosquito vectors of the genera *Aedes*, *Anopheles*, *Culex* or *Mansonia* are required for development of the larvae into the human infective stage, and for transmission to the human hosts. The vectors ingest MF during blood meals. In the insect, the larvae develop into infective larvae (L3), which are deposited on the skin of the humans during subsequent blood meals. The larvae enter the body through the wound made by the insect and undergo two more moults to develop into adult worms, completing the cycle.

The pathology that develops with LF has a spectrum of clinical states with two major poles. One pole is represented by microfilaraemic patients with high parasite numbers and downregulated cell-mediated immune responses, and the other by patients with LE or hydrocele (swelling of the scrotal/groin area), who typically have few or no parasites but vigorous specific immune reactions (3). LE and hydrocele are not mutually exclusive and both involve dilation of the lymphatic vessels and extravasation of fluid from the vessels into the surrounding tissues, indicating a shared pathogenesis. Among adult residents of endemic areas, 12·5% have clinical manifestations of LE and 21% of men have hydrocele (2), despite the fact that most individuals are presumably inoculated with L3 throughout life. Both pathologies develop progressively and not all affected individuals will progress to the most severe form of pathology.

The Global Programme for the Elimination of Lymphatic Filariasis (GPELF) strives to eliminate LF as a public health concern by 2020 by breaking transmission of the MF to uninfected persons in the community (http://www.filariasis.org) (4,5). The programme is conducted by endemic countries with the help of the WHO and numerous nongovernment organizations (NGOs) and undertakes annual administration of antimicrofilarial drugs to eligible members of the affected community, i.e. mass drug administration (MDA). In areas where the filarial nematode *Onchocerca volvulus* is also endemic, the drug combination of ivermectin (IVM, provided free to the WHO by Merck) and albendazole (provided free to the WHO by GlaxoSmithKline) is administered. In areas where only *W. bancrofti* or *Brugia* spp. is present, diethylcarbamazine (DEC, easily made in endemic countries) and albendazole (GlaxoSmithKline) are administered. Both IVM and DEC are effective at killing the MF, but have limited, long-term effects on the adult worms. For these reasons, the LYMFASIM programme, which models transmission and control of LF, predicts that administration of the drugs for at least 8 years, assuming a coverage of >65% of the affected population, is required (6).

With proper administration of the drugs and high coverage participation in the programmes, it is possible to achieve the desired block in transmission using MDA, as has occurred in some affected countries (7). This assumes that economic troubles or regional conflicts do not force the movement of endemic populations from areas with MDA coverage to areas without and that the worms do not develop resistance to IVM or DEC. However, apart from reducing new infection and therefore prevalence of pathology, the main aim of MDA, these microfilaricidal treatments do little for the ∼12·5% of infected individuals who suffer from LE or the 21% who suffer from hydrocele, as it is the death of the adult-stage worm that drives pathology (8). Currently, the MDA programmes to eliminate LF mainly offer hygiene and management courses to patients with this pathology. While beneficial to various extents, these methods do not greatly reverse the pathology and therefore may hinder compliance (9). Understanding the immunological, molecular and genetic causes of LE (and hydrocele) will help, as described below, to develop better methods of identifying persons at risk, and preventing/ameliorating these severe pathologies, thus improving the daily lives of the affected individuals.

## Clinical findings

The presence of adult worms in the lymph vessels and lymph nodes is seen as a trigger for pathology. Histologically, there is little reaction around adult worms as long as they are alive, but inflammation occurs when worms die, either drug-induced or spontaneously (8). Granulomas arise around those worms, characterized by macrophages which develop into giant cells; as well as plasma cells, eosinophils and neutrophils. The clinical symptom is filarial fever that starts at the location of worm death and typically leads to retrograde lymphangitis (painful, with swelling) and lymphadenitis, which lasts for approximately 1 week during which the patient is immobilized. The usually abrupt end of the fever is accompanied by scaling off of the skin. While these episodes are clinically transient in most infected individuals, they can be the starting point for more chronic pathology leading to lymph fibrosis, particularly in genetically predisposed individuals whose immune system tends not to be appropriately downregulated, but is rather stimulated (see below). However, subclinical changes, such as lymph vessel dilation, are almost invariably found in all infected individuals, e.g. in the scrotal area of adult men infected with *W. bancrofti* (*Brugia* does not reside in the scrotal area), where they can be detected by ultrasonography (10) [grading of dilation is described by Debrah *et al.* (11), see their Figure 9].

Lymph vessel dilation, not obliteration, is probably the early event following antigenic stimulation, which takes place while the adult worms are still alive, i.e. when offspring larvae are being released. Many of these larvae are degenerate and will be taken up by phagocytic cells; it is known that exposure of phagocytes to filarial antigens is accompanied by triggering of the innate immune system (12–14), with the release of not only pro-inflammatory cytokines but also of molecules that promote lymphangiogenesis, as detailed below. In addition to antigens from the worm itself, those from its *Wolbachia* endosymbionts appear to play a major role in pathogenesis, as their depletion by doxycycline leads to a reduction of elevated plasma levels of lymphangiogenesis factors, which precedes the reduction of lymph vessel dilation [(11); see in detail below].

The enlarged lymph vessels become less efficient at transporting lymph from the periphery, which in the legs is always oriented against gravity, making the system more vulnerable to exogenous microorganisms, which may enter the lymphatics following minor injuries that go unnoticed in people without lymphatic disease. However, in people with altered lymph vessels these secondary infections trigger more inflammation/lymphangiogenesis, presenting mainly as acute (bacterial) dermatolymphangioadenitis (ADLA) (15–17). Insufficient fluid transport will lead to fluid extravasation, particularly in the lower limbs, and eventually to LE. Thus, exogenous microorganisms, in particular bacteria of the genus streptococci, are important for the progression of the condition from subclinical lymphatic aberrations to LE, for which Dreyer and colleagues (15) have defined seven stages (stages are also described in Ref. (11)]. This is different from the second most frequent form of pathology, hydrocele, i.e. accumulation of fluid between the two folia of the tunica vaginalis in the scrotum. In this condition, lymph vessel enlargement and later obliteration, probably including a chronic inflammatory stimulus for the tunica vaginalis to secrete fluid between the two folia, lead to the pathology independent of exogenous bacteria. To quantify improvement/worsening of hydrocele, a staging system using ultrasonography of the scrotal area has been developed (18).

## Immunological findings

Neonatal tolerance has been recognized as a major factor that prevents pathology following filarial infection (19). This became evident when two scenarios in which nonendemic persons exposed to filarial infection were analysed. First, of the >38 000 US Naval personnel with LF exposure in World War II in the South Pacific, >10 000 (27%) had clinical signs of filarial fever, while only 20 (!) individuals actually became microfilaraemic. While this was a rather short-term exposure and did not lead to chronic pathology, permanent resettlement of people from nonendemic into highly endemic areas did, as observed in Indonesia (20); this was also accompanied with a high prevalence of clinical signs and low prevalence of microfilaraemia. In sharp contrast, the majority of individuals *born* in endemic areas are chronically infected and many of them are microfilaraemic, without clinical signs of pathology. In long-term studies, the tendency to develop asymptomatic infection with high parasite loads and microfilaraemia was associated with the status of being born from microfilaraemic mothers (21); this earlier groundbreaking study from a Pacific island has recently been confirmed by a prospective analysis from Kenya (19,22).

The molecular mechanisms associated with this neonatal tolerance were found to be associated with markers of immunosuppression and/or immune tolerance to filarial antigens. However, not all individuals born in endemic areas acquire this status. This is probably due to a mixture of environmental factors (transmission level, co-infections) and genetic propensity (see below). The general picture is that lack of filarial-specific T-cell proliferation and production of IL-10 or TGF-β is associated with the permissive status, while a prospectively lower likelihood to become infected in an endemic area correlates with higher levels of IFN-γ, IL-5 and IL-13 (19,22,23), and individuals with pathology may in addition show a tendency to react with higher IL-17 responses to exogenous stimuli (24). In accordance with the finding that IL-10 promotes IgG4 responses (25), whereas Th2 reactions induce IgE, patients with the permissive status show higher levels of IgG4 than do those with pathology (3).

Raised levels of IL-6 and IL-8 emerged as markers of acute as well as chronic disease in a large-scale study in India (26). Whether this is the cause or rather the consequence of chronic disease is not fully clear. In either case, the release of markers of the innate immune response is at least in part due to the reactions against bacterial products, i.e. those from the *Wolbachia* endosymbionts, as could be shown *in vitro* and *in vivo* in several studies (12–14). In support of the innate markers being the cause rather than the consequence/epiphenomenon of pathology, they decrease in serum following depletion of *Wolbachia* by treatment with doxycycline (27), and this *precedes* improvement of pathology such as lymph vessel dilation (see the section on *Wolbachia* below).

Common sequelae of the stimulation of the innate response include the production of vascular endothelial growth factors, e.g. by macrophages [reviewed in Ref. (28)]. These factors are elevated following LF infection, with different levels of expression in the different disease forms (see below). They seem to be instrumental in the stimulation of lymph vessel growth, which is the first step in a series of events that leads to the different lymphatic pathologies, described below.

Recent studies focusing on molecular mechanisms regulating blood and lymphatic vessel growth (29–31) have shown that vascular endothelial growth factors (VEGF) control angiogenesis and lymphangiogenesis in humans (32–35). The VEGF family comprises five members (36), including VEGF-A, a potent regulator of normal and abnormal angiogenesis (37). Over-expression of VEGF-A in the skin of mice results in increased vascular density, enhanced leucocyte adhesion, and tissue infiltration and also promotes lymphangiogenesis (38). VEGF-C stimulates the migration of endothelial cells and increases vascular permeability and lymph endothelial proliferation, but at higher concentration than that of VEGF-A (39). Over-expression of VEGF-C in the skin of transgenic mice results in lymphatic endothelial proliferation and dilation of lymph vessels (40). Signals for endothelial cells are mediated through VEGFR-2 in blood vascular endothelial cells, and generally via VEGFR-3 in the lymphatic endothelial cells (29,41).

The expression of VEGF-A and VEGF-C has been shown to be up-regulated by pro-inflammatory cytokines, such as IL-1β, TNF and IL-17 (42), suggesting that pro-inflammatory cytokines affect the lymphatic vessels via VEGF-A and VEGF-C (43,44). Major inducers of pro-inflammatory mediators are the *Wolbachia* essential endosymbionts of filariae.

## Role of *Wolbachia*

It has been known for more than 30 years that filarial nematodes contain the endosymbiotic bacteria *Wolbachia* of the order Rickettsiales (45). These endosymbiotic bacteria are found in the hypodermis of male and female worms, in the oocytes, embryos and larval stages (46). As in many animal filarial species, these endobacteria are present in human filariae including *W. bancrofti*, *Brugia* species and *O. volvulus* (4,47–49), with the exception of a few species such as *Loa loa* (50,51). The discovery of the essential role of *Wolbachia* in worm fertility and survival has resulted in the development of an antifilarial chemotherapy with doxycycline, which depletes *Wolbachia* from the worms and leads to long-term worm sterility (47,48) and macrofilaricidal activity in both bovine (52) and human onchocerciasis (53) as well as human LF (11,54,55).

In addition to the possible role of *Wolbachia* as a chemotherapy target, it has become clear that *Wolbachia* antigens can stimulate host immune responses that may be associated with development and progression of pathogenesis of filarial diseases. Low level exposure of the immune system to *Wolbachia* stimuli could occur via the uptake of degenerate larvae released by the females by phagocytic cells. Upon death of the MF, e.g. after DEC or IVM treatment, or natural death of an adult worm, the immune system would be exposed to a large amount of pro-inflammatory stimuli, including large numbers of *Wolbachia.* In a laboratory model of onchocerciasis for instance, *Wolbachia* were shown to mediate neutrophil infiltration and keratitis (blindness) when a worm extract including *Wolbachia* antigens was injected into the eyes of mice (13,56). Such a reaction is not seen with *Wolbachia*-depleted extract because during antibiotic treatment, the dying/dead endobacteria are cleared from the worm probably via a normal mechanism for maintaining the symbionts at levels acceptable to nematode survival. This clearance, which occurs slowly, presumably also prevents the exposure of the immune system to endobacterial ligands either by degradation or by modification.

*Wolbachia* have been shown to stimulate pro-inflammatory cytokines, such as TNF, IL-1β and IL-6, and nitric oxide (12–14), which are known to upregulate the expression of VEGF-C (43,44). This stimulation is mainly signalled via TLR 2, which in other inflammatory diseases is known to mediate the upregulation of VEGFs (57). This raises the possibility that pro-inflammatory cytokines such as TNF and IL-1β induced by *Wolbachia* could affect lymphatic vessels by induction of VEGFs. Indeed, elevated serum levels of VEGF-A and endothelin-1 (58) as well as VEGF-C and VEGFR-3 (11) were found in filaria-infected patients.

It is therefore conceivable that any therapeutic intervention that causes reduction of lymphangiogenic factors may lead to reduction of dilated lymphatic vessels. Evidence was provided in a recent study, which showed that increased levels of VEGF-C were associated with infection, while VEGFR3 levels were yet significantly higher in LE than in other conditions of LF infection (11). Doxycycline treatment not only reduced *Wolbachia* copy numbers by 95% in *W. bancrofti*-infected patients but also reduced the plasma levels of VEGF-C/sVEGFR-3, resulting in amelioration of dilated lymphatic vessels and improvement in the conditions of LE patients. Affected legs of all the doxycycline-treated patients reverted significantly to a lower stage at 12 months post-therapy, while the condition of the placebo-treated patients stayed the same or increased to a higher stage (11). However, the improvement was more pronounced in patients with initial lower stages of LE (stages 1–3) than those with higher stages [stages 4–6; staging according to Dreyer *et al.* (59)]. Importantly, the reduction of the VEGF-C/sVEGFR-3 preceded the amelioration of the dilated lymphatic vessels and LE. Because all participants received IVM 4 months after doxycycline treatment to clear the MF from their blood, it was concluded that this effect was attributable to doxycycline, and thus associated with reduction of *Wolbachia*.

Data suggest a similar phenomenon for hydrocele being associated with lymphangiogenic markers, specifically VEGF-A (54). In hydrocele patients, targeting the *Wolbachia* endosymbionts by doxycycline led to a reduction in plasma levels of VEGF-A, with consequential amelioration of the size of hydrocele (60). Importantly, the amelioration and VEGF-A reduction was observed in patients who were circulating filarial antigen (CFA)-positive (i.e. actively infected), but not in doxycycline-treated patients who were CFA-negative or in the placebo patients. These data are in agreement with the hypothesis that progression of infection to hydrocele may be contributed to by the over-expression of vascular and lymphangiogenic factors such as VEGF-A, which promotes extravasation of fluid and plasma proteins, including fibrin from the blood vessels into surrounding tissues. Secretion of VEGF-A molecules in the scrotal region of hydrocele patients could be responsible for: (i) extravasation and accumulation of fluids, plasma, lymph, etc. from the blood and lymphatic vessels into the scrotal region, resulting in the formation of hydrocele, chylocele and lymphocele; (ii) formation of nodules in hydrocele patients which are usually observed just before hydrocele development when DEC is administered (61). The formation of these nodules might result from the death of adult worms due to DEC treatment, which would in turn lead to liberation of VEGF-A, in part due to release of *Wolbachia.* However, in a study conducted by Esterre *et al.* (58), the level of serum VEGF was shown to remain the same in patients with bancroftian filariasis after DEC treatment. While more research will be needed to elucidate the reasons for this lack of VEGF increase, one might conclude that this may be due to the fact that DEC treatment has no effect on bacteria (neither *Wolbachia* nor exogenous species that augment and maintain the pathology of LE (15), and therefore has no effect on pro-inflammatory cytokines that regulate this VEGF family, although it does partially reduce adult worm levels*.*

Although further investigations are needed to elucidate the mechanism of doxycycline in reducing lymphangiogenic factors and lymphatic vessel dilation, our data suggest that plasma levels of VEGF-A/VEGF-C/sVEGF-R3 may correlate with disease progression in LF leading to LE and hydrocele, and hence, might be used as prognostic indicators of an increased risk of LF pathology before it manifests. The data also indicate that filarial pathology may be caused by over-expression of lymphangiogenic factors as a result of stimulation of pro-inflammatory cytokines induced by the filarial and *Wolbachia* antigens in the lymphatic vessels. In further support of this in an animal model of filariasis, VEGF gene expression following infection with L3 can be readily detected (Specht S. and Hoerauf A., unpublished data), suggesting an important role of VEGFs for the establishment of L3 in the host’s body.

## Role of the nematode

In addition to their endobacteria, filarial nematodes themselves are a source of ligands which can induce an immune response that might lead to the development of LE. As has been shown, macrophages and monocytes produce pro-inflammatory cytokines in response to protein extract from *Wolbachia*-depleted *B. malayi* or *Acanthocheilonema viteae*, a filarial nematode without endosymbionts (14,56). However, comparison of the *Wolbachia*-containing extract with the reaction produced by *Wolbachia*-containing extract reveals that the level of induction from the nematode is greatly reduced. Such a reduced reaction makes sense for the long-term survival of the nematodes as it is known that MF induce a hyporesponse (23,62–65). Two studies that examined the effect of doxycycline treatment prior to antifilarial treatment also demonstrated that the nematodes themselves can induce a pro-inflammatory response. In these studies, adverse reactions to IVM or DEC treatment were monitored in patients who were given doxycycline or placebo prior to treatment. When MF were depleted of *Wolbachia*, adverse reactions still occured, but they were less severe (27,66). Thus, under normal conditions when the endobacteria are contained within their nematode hosts and separated from the immune system, few cytokines that could induce VEGF or other factors involved in LE development are released. However, upon death of the MF or adult worms, large amounts of immune stimulatory ligands are quickly released, including the highly active *Wolbachia* components, which may then induce a strong pro-inflammatory response.

## Immunogenetics

As explained above, host immune responses are required for controlling worm load at the level of both circulating larvae and adult worms in the mammalian host. These factors can then induce the expression of other genes, such as VEGFs, that are hypothesized to contribute to LE. The immune responses differ between individuals who have pathology and those who do not, even if the parasitological parameters are similar. One explanation for different responses of patients is that single-nucleotide polymorphisms (SNPs) alter the expression of or the activity of immune factors, leading to the broad range of disease manifestations that can develop.

Studies have shown that susceptibility to infection and parasite load cluster in families (67,68). One of the first studies dealing with genetics as a factor for susceptibility to filarial infection was carried out in a Polynesian population infected with *W. bancrofti*. The results showed that 52% of the children whose parents both had LF were also infected. This dropped to 18% when just one of the parents had LF, and was 0% when neither parent had LF. A gene frequency of 0·82 was estimated for the unidentified allele in this study (67). Two studies in Indonesia have also shown family clustering of *B. malayi* infection. Both studies measured microfilaraemia and antibodies to *B. malayi* as markers of infection. Both markers were found to cluster in families, independent of household and environmental factors (69,70).

There are also published studies on the genetic basis for developing LE pathology in lymphatic filariasis. Two studies in Haiti dealing with the genetic basis for developing LE found that 42% of the patients with LE in at least one leg also had parents with LE. The incidence of multiple cases of LE was also clustered in families (71,72). Studies on the exact genetic factors that lead to families having a greater incidence of filariasis and LE have only recently begun by dealing with SNPs.

Several studies have described an association of SNPs with either susceptibility to disease or pathology in LF. Choi *et al.* have shown in a study in South India that patients are more susceptible to *W. bancrofti* infection if they are homozygous for the allele that is responsible for lower levels of chitotriosidase (CHIT1), a protein found in phagocytic cells (73). Interestingly, no association of susceptibility to infection with CHIT1 was found in Papua New Guinea (74), demonstrating the importance of genotyping populations from different endemic areas.

Recently, we have published a case–control study that examined the role of VEGF-A SNPs in hydrocele, pathology with some similarity to LE in that both the dilation of the lymph vessels and extravasation of lymph into the surrounding tissues are a shared sequela. In the study, three promoter SNPs were tested for association with hydrocele. The genotype frequency of the −460 C/T polymorphism differed significantly between patients with and without hydrocele. The allele C, known to upregulate VEGF-A production, was significantly higher in hydrocele patients (74%) than in nonhydrocele LF patients (4%). A positive correlation was also observed between plasma concentrations of VEGF-A and the stage of hydrocele, providing evidence that the −460 C polymorphism is a novel genetic risk factor for hydrocele development in lymphatic filariasis (18).

While similar studies have not yet been published on immunogenetic factors and their role in LE, the preceding SNP results support a causal role of patient genetics in LF development and specifically identify immunological markers having a causal role in filarial pathology. Similar to what has been shown for primary LE (1,75), there is a genetic component to the development of secondary LE in response to filarial infections. To date, most publications have used a candidate gene approach to find the association of SNPs with disease susceptibility or pathology. Our own studies have found an association of IL-18 and TLR-4 SNPs with LE in *W. bancrofti* and *B. timori* infections. Additionally, we have found an association of IL-4 receptor and TGF-beta SNPs with microfilaraemia and latent infection, respectively (Debrah A., Pfarr K., Hoerauf A., Albers A., Becker T., Mand S., unpublished observations). VEGF-A is also likely to play a role in LE development because of its role in lymphangiogenesis (60), as has been shown for hydrocele (18). The next step will be to conduct a genome-wide scan to identify loci and genes associated with LE and hydrocele. To this end, we have completed recruitment of unrelated patients in Ghana for a case–control study.

## Ongoing studies and future assessments

Knowledge of genomic loci linked to parasite loads and pathology would help in the understanding of pathogenesis which could lead to the development of strategies, beyond the supportive care advocated for hydrocele and LE (11,18), to ameliorate pathology in the 40 million people affected by LE. Such strategies would in turn help in compliance with the current drug therapies to interrupt transmission, as the inability to treat the physical pathology is a key factor in noncompliance (9). Knowing genetic markers for LE and hydrocele could also provide a way to identify persons at risk before pathology is evident and might become the basis for development of a screening test that, in the case of LE, which develops early in life, might be applied to school-aged children.

From the studies using doxycycline treatment, it is clear that more research is needed to unravel the associations between *Wolbachia*, lymphangiogenic molecules, lymphatic dilation and pathology development. For doxycycline to exert its ameliorating activity by targeting *Wolbachia*, it is mandatory that the targets, i.e. adult worms, MF or at least incoming L3/4 larvae, are present in the host’s body. Although our results indicate that the depletion of *Wolbachia* was the most probable cause of the reduction in LE, it could not be ruled out that doxycycline had another target, either the host lymphatic endothelial cells, or another bacterial or fungal infection, which led to these astounding results. It is important, therefore, to investigate whether the targeting of either *Wolbachia* or other opportunistic bacteria is the essential mechanism for the improvement of LE observed. To ascertain this fact, there is an ongoing study (ISRCTN No. 90861344) with three different treatment regimens: one group treated with doxycycline, targeting *Wolbachia* and other exogenous bacteria; one group treated with amoxicillin, targeting only exogenous bacteria with no effect on *Wolbachia* and the third group treated with placebo. To date, there has been significant amelioration in the doxycycline vs. placebo groups and also between doxycycline and amoxicillin groups, while there was no significant amelioration in the amoxicillin vs. placebo groups, suggesting that targeting of *Wolbachia* by doxycycline might be more beneficial than targeting potential opportunistic exogenous bacteria. Final assessments are still pending, but these trials show that there is a new hope for LE and hydrocele patients.

Another factor to consider is the direct effects of doxycycline that could lead to reduced pathology. Recently, Fainaru *et al.* showed that oral doxycycline prevents VEGF-mediated vascular permeability and IL-2-induced pulmonary oedema (76). At the molecular level, doxycycline appeared to act on the adherence junction in the vasculature with increased expression of VE-cadherin, thus maintaining the cell junctions between endothelial cells and reducing their permeability.

Antiwolbachial therapy, especially with doxycycline, would be more effective than therapy with other antifilarial drugs because it depletes *Wolbachia* which leads to a reduction in pro-inflammatory stimuli that in turn can induce VEGF; eventually kills adult worms, removing another source of pro-inflammatory stimuli; and may directly act on the lymphatic cells to maintain cell junctions and prevent extravasation of fluid into the surrounding tissue. Hence, doxycycline treatment has a good chance to be the first chemotherapeutic approach to address lymphatic pathology, especially in patients with early-stage LE which is easier to reverse. Patients with LE suffer considerably, and may reasonably be expected to take doxycycline for 6 weeks without the need of a directly observed treatment (DOT). Thus, doxycycline is a drug immediately available to physicians that will allow them to better treat their patients who come to them with the goal of improving their physical condition.
